# *Yersinia enterocolitica*, a Neglected Cause of Human Enteric Infections in Côte d’Ivoire

**DOI:** 10.1371/journal.pntd.0005216

**Published:** 2017-01-12

**Authors:** Daniel Saraka, Cyril Savin, Stephane Kouassi, Bakary Cissé, Eugène Koffi, Nicolas Cabanel, Sylvie Brémont, Hortense Faye-Kette, Mireille Dosso, Elisabeth Carniel

**Affiliations:** 1 Environnement and Health department, Institut Pasteur, Abidjan, Côte d'Ivoire; 2 Yersinia Research Unit and National Reference Laboratory, Institut Pasteur, Paris, France; 3 Bacteriology and Virology department, Institut Pasteur, Abidjan, Côte d'Ivoire; Fondation Raoul Follereau, FRANCE

## Abstract

**Background:**

Enteropathogenic *Yersinia* circulate in the pig reservoir and are the third bacterial cause of human gastrointestinal infections in Europe. In West Africa, reports of human yersiniosis are rare. This study was conducted to determine whether pathogenic *Yersinia* are circulating in pig farms and are responsible for human infections in the Abidjan District.

**Methodology/Principal findings:**

From June 2012 to December 2013, pig feces were collected monthly in 41 swine farms of the Abidjan district. Of the 781 samples collected, 19 *Yersinia* strains were isolated in 3 farms: 7 non-pathogenic *Yersinia intermedia* and 12 pathogenic *Yersinia enterocolitica* bioserotype 4/O:3. Farm animals other than pigs and wild animals were not found infected. Furthermore, 2 *Y*. *enterocolitica* 4/O:3 strains were isolated from 426 fecal samples of patients with digestive disorders. All 14 *Y*. *enterocolitica* strains shared the same PFGE and MLVA profile, indicating their close genetic relationship. However, while 6 of them displayed the usual phage type VIII, the other 8 had the highly infrequent phage type XI. Whole genome sequencing and SNP analysis of individual colonies revealed that phage type XI strains had unusually high rates of mutations. These strains displayed a hypermutator phenotype that was attributable to a large deletion in the *mutS* gene involved in DNA mismatch repair.

**Conclusions/Significance:**

This study demonstrates that pathogenic *Y*. *enterocolitica* circulate in the pig reservoir in Côte d'Ivoire and cause human infections with a prevalence comparable to that of many developed countries. The paucity of reports of yersiniosis in West Africa is most likely attributable to a lack of active detection rather than to an absence of the microorganism. The identification of hypermutator strains in pigs and humans is of concern as these strains can rapidly acquire selective advantages that may increase their fitness, pathogenicity or resistance to commonly used treatments.

## Author Summary

Diarrhea is a major public health problem in developing countries, especially in Africa, but the causative agents are often unknown, impeding the implementation of appropriate therapeutic measures. Although pathogenic *Yersinia enterocolitica* are a frequent cause of gastroenteritis in developed countries, reports of human yersiniosis are scarce in West Africa. We performed this study to determine whether this pathogen was present in pigs (the natural reservoir of this bacterium) in various swine farms of the Abidjan district, and whether it was causing human gastro-intestinal infections. We show here that this bacterium is indeed circulating among Ivorian pigs and is causing human digestive disorders with a frequency similar to that reported in developed countries. The paucity of reports of this infection in African countries may be explained by the difficulty of isolating *Y*. *enterocolitica* from stools and the need for specific and time-consuming procedures. During this study we also made the unexpected observation that some Ivorian strains have acquired the capacity to mutate at a much higher frequency than normal strains. This property may render them better fitted to new environments, more virulent to their host, or capable of resisting some commonly used treatments, which could be of great public health concern.

## Introduction

*Yersinia enterocolitica* is an enteropathogenic bacterium responsible for human gastroenteritis. This species belongs to the genus *Yersinia*, and to the family *Enterobacteriaceae*. Clinical presentation of yersiniosis includes diarrhea, abdominal pain, fever, and sometimes vomiting [1]. Although the infection is often mild and self-limiting, more severe clinical presentations such as pseudo-appendicular syndromes mimicking an appendicitis [2] or septicemia in elderly and immuno-compromized patients [3] can occur. Reactive arthritis and erythema nodosum are the most frequent secondary complications [4].

Enteric yersiniosis is a foodborne disease [5,6], which is transmitted through the fecal-oral route. The species *Y*. *enterocolitica* is subdivided into 6 biotypes [7]. Biotype 1A is non-pathogenic while the 5 other biotypes (1B, 2–5) cause human and/or animal infections. The biotype the most frequently responsible for human infections worldwide is biotype 4 [8], which is almost systematically associated with serotype O:3 (4/O:3), followed by bioserotype 2/O:9. Pigs are the main reservoir of bioserotype 4/O:3 strains [8]. These animals are asymptomatic carriers of the bacteria in their tonsils and intestinal tract, and they shed the enteropathogen in the environment with their stools. Contamination of pork meat often occurs during pig evisceration at slaughter [9].

Although *Y*. *enterocolitica* represents the third cause of bacterial diarrhea in Europe, after campylobacteriosis and salmonellosis [10] reports of human yersiniosis are scarce in West Africa. A systematic survey of children with diarrhea in Ouagadougou (Burkina Faso) allowed the isolation of *Yersinia* strains from 1.7% of the stools tested [11]. However, no indication of the species or biotype of the strains was provided, making difficult to estimate whether these isolates corresponded to saprophytic or pathogenic species. *Y*. *enterocolitica* strains were also isolated from the stools of children with gastroenteritis in Gabon, but their bioserotype was not determined [12]. In Ghana, a few *Y*. *enterocolitica* strains were isolated from blood products at transfusion centers [13] or were detected by PCR in the blood of some patients with sepsis [14], indicating that severe cases of yersiniosis occur in this country. The most active West African country for the survey of *Yersinia* infections is Nigeria. Several studies reported the isolation of pathogenic *Y*. *enterocolitica* from the stools of patients presenting with enteric infections [15–19].

An active surveillance of the animal reservoir also revealed the presence of pathogenic bioserotypes of *Y*. *enterocolitica* pigs, bovine and sheep in Nigeria [20–23]. In contrast, a survey of the pig reservoir in Senegal [24] and Burkina Faso [25] did not identify any *Yersinia* strains. The search for these bacteria in water sources in Nigeria also remained negative [26].

These data suggest that *Y*. *enterocolitica* is present in the animal reservoir and causes human infections in West Africa. Insufficient public health surveillance and inappropriate isolation procedures may account for the paucity of reports of this infection [27]. The fast-growing demand for milk and meat in urban centers in resource-limited countries is leading to the intensification of livestock production systems, especially in peri-urban areas. However, because efficient zoonosis surveillance and food safety are lacking, the risk for zoonosis transmission is increasing, particularly in rapidly growing urban centers of resource limited countries [28].

In Côte d'Ivoire, there is some indication that *Y*. *enterocolitica* circulates and may be a cause of human infections. In 1983, a search for the presence of fecal coliforms in drinking well water and in human stools allowed the isolation of a few *Yersinia* strains [29]. As their species were not determined, whether they were environmental non-pathogenic strains, or enteropathogens could not be established. More recently, three *Y*. *enterocolitica* strains of bioserotype 4/O:3 were isolated from pig carcasses at slaughter [30]. These results thus showed the presence of pathogenic *Yersinia* in the pig reservoir, suggesting that *Y*. *enterocolitica* may be a cause of human gastroenteritis in Côte d'Ivoire.

This study aimed at further investigating the carriage of *Y*. *enterocolitica* in the animal reservoir and at estimating the prevalence of enteropathogenic *Yersinia* in human diarrheal diseases in the Abidjan area of Côte d'Ivoire.

## Methods

### Ethics statement

The human and animal components of this study were approved by the National Ethical Research Committee of Côte d’Ivoire (Ref 095/MSLS/CNER-kp). Written informed consents were obtained from the patients or their parents, by medical doctors and laboratory teams in charge of surveillance activities, and from farmers and animal owners.

### Sample processing

This study was carried out from June 2012 to December 2013. Forty-one swine farms distributed in 4 sub-prefectures of the Abidjan district (Abidjan, Anyama, Bingerville and Songon) were selected based on their high pig production capacity and were visited monthly. Two to 3 stool samples were collected by rectal swab from apparently healthy pigs randomly sampled in each farm and pooled. Fecal samples were also taken from cattle in the infected farms and pooled. Wild animals (rats and gigantic snails) living around the farms were also captured and their intestinal contents were individually analyzed for the presence of *Yersinia*. Fecal samples were collected from humans with digestive disorders in 8 hospitals from the 4 sub-prefectures. Animal feces and intestinal contents, as well as human stools were collected freshly and stored cold during transportation in an ice box to the laboratory for immediate processing.

### Isolation, identification and characterization of *Yersinia* strains

*Yersinia* strains were isolated using two stages enrichment procedures as described by the Department of Food and Environmental Hygiene in Finland [31]. This procedure included prior-enrichment at 25°C during 24h of 1 ml of sample in 9 ml of Brain Heart Infusion broth containing 2.5 mg/l novobiocin. This was followed by an enrichment step for 7 and 14 days at 4°C in a modified phosphate buffer saline supplemented with 1% mannitol, 0.15% bile salts, and 0.5% soy peptone. An alkali treatment for 20s of 0.5 ml of the enriched sample in 4.5 ml of 0.25% potassium hydroxide was then performed to reduce the background-contaminating flora as described [32]. A 10 μl volume of the enriched sample was then streaked on Cefsulodin-Irgasan-Novobiocin agar (CIN). The plates were incubated under aerobic conditions at 30°C for 18h–48h. Putative *Yersinia* colonies were identified with oxidase, Kligler iron and Christensen’s urea. Oxidase-negative, glucose-positive, H_2_S-negative and urease-positive colonies were finally identified with API 20E strips. Colonies displaying typical patterns were further characterized at the French *Yersinia* Reference Laboratory (Institut Pasteur, Paris) for species determination, biotyping and serotyping [3]. Phage typing was performed and interpreted as described in [33], except that phage type VIII was characterized by a susceptibility to all phages except phage l (observation from the *Yersinia* Reference Laboratory).

### Antibiotic susceptibility testing

From an 18-24h bacterial culture onto trypticase soy agar, a bacterial suspension in saline was prepared at McFarland 0.5 (equivalent to ≈10^8^ cfu/ml). Antibiotic susceptibilities were determined by the disc diffusion method on Mueller-Hinton agar (Oxoid) according to the procedure described in [34]. The results were interpreted according to the guidelines of the European Committee on Antimicrobial Susceptibility Testing (EUCAST: http://www.eucast.org). The antimicrobial drugs tested and their concentrations on the discs (BioRad) were the following: amoxicillin (25 μg), amoxicillin-clavulanic acid (20 μg/10 μg), cefalotin (30 μg), cefoxitin (30 μg), ceftriaxone (30 μg), ciprofloxacin (5 μg), nalidixic acid (30 μg), trimethoprim (5 μg), sulphonamide (200 μg), tetracycline (30 UI) and ticarcillin (75 μg).

### Primers and PCR conditions

Bacterial suspensions from 8-24h bacterial cultures were diluted to 10^4^ cfu/ml in sterile water (Eurobio) and centrifuged for 10 min at 13,300 x g at 4°C. Genomic DNA was extracted using the phenol/chloroform and alkali lysis methods [35]. Primers specific for the chromosomal genes *ail* (attachment invasion locus) and *ystA* (*Yersinia* heat-stable enterotoxin), and for the pYV plasmid-borne gene *yadA* (*Yersinia* adhesin) of *Y*. *enterocolitica* were used (S1 Table). PCR reactions were carried out in a volume of 50 μl containing 2.5 μl of 5X Green Flexi buffer (Promega), 2.5 μl of 5X Colorless Flexi buffer 3 μl of 25 mM MgCl2; 1 μl of 10 μM nucleotides dATP, dTTP, dGTP and dCTP; 1 μl of a 20 μM solution of each primer (S1 Table); 0.2 μl of 5 U/μl Taq DNA polymerase and 5 μl of DNA. The amplifications were performed in a thermal cycler with the following conditions: denaturation at 94°C for 5 min, followed by 40 cycles of denaturation at 94°C for 30s, annealing at 60°C for 30s, and extension at 72°C for 1 min, with a final extension at 72°C for 5 min. PCR products (10 μl) were subjected to electrophoresis in a 2% agarose gel and stained with ethidium bromide.

### Genotyping

Pulsed Field Gel Electrophoresis (PFGE) of *Y*. *enterocolitica* 4/O:3 isolates was carried out as previously described [36]. The genomic DNA was digested with *Pme*I and *Spe*I and subjected to electrophoresis for 24h using pulse times ranging from 1 to 13 s at 14°C for *Pme*I, and from 1 to 15 s at 17°C for *Spe*I, with an angle of 120° in a CHEF-DR III apparatus A middle-range PFGE size marker) was used. MLVA **(**Multiple-locus variable-number tandem-repeat analysis) was based on six loci previously defined [37] and was performed by sequence analysis of the PCR products. The PCR mixtures (40 μl) contained 100 ng of DNA template, 0.2 μM of each primer, 1.25 unit of Taq DNA polymerase (Thermo Scientific), 200 μM of dNTPs, 1.25 mM MgCl2, and a final concentration of 1X Taq buffer. The amplification was carried out in a DNA thermocycler with a pre-denaturation step at 94°C for 10 min, followed by 35 cycles of denaturation at 94°C for 30 s, annealing at 58°C for 30 s, and elongation at 72°C for 30s. A final 3 min elongation step at 72°C was done after the last cycle to ensure complete amplicon extension. PCR products were sequenced, and the number of repetitions for each locus was determined by the analysis of each sequence.

### Whole genome sequencing and analysis

Two ml of overnight bacterial cultures at 28°C were centrifuged at 5,000 x g for 5 min, and the genomic DNA was extracted from the cell pellet using the Genomic DNA mini kit. The DNA was suspended in 100 μl of elution buffer and quantified with the LUX (Thermo Fisher Scientific). Genomic libraries were prepared with 0.1 ng of DNA as template, using the Nextera XT protocol on the SureCycler 8800 thermal cycler (Agilent). The libraries were purified with the AMPure beads and quality control was performed with the High Sensitivity D1000 kit on Tape station 2200 Inserts were sized (400–900 bp) using the Pippin Prep kit CDF 1510 and enriched with 35 cycles of qPCR with the KAPA kit on the Lightcycler 96 before library quantification and validation. Hybridization of the library to the flow cell and bridge amplification was performed to generate clusters. Paired-end reads of 150 cycles were collected on a NextSeq500 sequencer using the HighOutput kit.

Demultiplexing and generation of FASTQ files from the raw sequence data were performed using the bcl2fastq software. Trimming, clipping, and filtering off exogenous and/or non-confident bases (options:–q 13, -l 30, -p 80) within FASTQ files were performed with AlienTrimmer [38]. Redundant or over-represented reads were reduced using the khmer software package (option:–c 70) [39]. Finally, sequencing errors were corrected using Musket [40] and overlapping paired reads were merged with FLASH [41]. A *de novo* assembly was performed for each strain with the quality-filtered reads using SPAdes v3.6 (options:-k 21,33,55,77—only-assembler—careful) [42].The alignment of the quality-filtered reads against a reference genome YE1203 (accession number: HF933425) was performed using BWA v0.7.7, and variant calling (SNPs and indels) using SAM tools v1.2 and VarScan v2.3.6 (options:–min-coverage 30 and–min-var-freq 0.8) [43].

Genome sequences are available in European Nucleotide Archive under BioProject PRJEB13626 (sample ID starting with ERS and accession numbers starting with FK) for strains IP134 (ERS1122523, FKKS01000001-FKKS01000141), IP35459 (ERS1122524, FKKM01000001-FKKM01000146), IP35462 (ERS1122525, FKKN01000001-FKKN01000154), IP35463 (ERS1122526, FKKL01000001-FKKL01000153), IP35464 (ERS1122527, FKKW01000001-FKKW01000150), IP35465 (ERS1122528, FKKU01000001-FKKU01000159), IP35466 (ERS1122529, FKKJ01000001-FKKJ01000148), IP35467 (ERS1122530, FKKT01000001-FKKT01000151), IP35470 (ERS1122531, FKKV01000001-FKKV01000149), IP35471 (ERS1122532, FKKP01000001-FKKP01000150), IP35472 (ERS1122533, FKKQ01000001-FKKQ01000151), IP35474 (ERS1122534, FKKO01000001-FKKO01000149), IP35475 (ERS1122535, FKKK01000001-FKKK01000157), IP35477 (ERS1122536, FKLN01000001-FKLN01000190), IP35478 (ERS1122537, FKKR01000001-FKKR01000152).

### Hypermutator phenotype

Strains were streaked on TSA agar plates. Ten colonies were picked and were grown in 10 ml Luria Bertani (LB) broth under agitation at 28°C for 24h. OD_600_ values were recorded and 200 μl of each culture were plated onto LB agar containing 50 μg/ml nalidixic acid or 100 μg/ml rifampicin.All plates were incubated at 28°C for 3 days. Colony enumeration was performed with a Scan500 automatic colony counter. The results were expressed as the number of colonies on antibiotic plates per 10^9^ cfu of the original inoculum.

## Results

### Search for the presence of *Yersinia* in pig feces

A total of 781 samples of pooled pig feces collected over the 19 months study period in 41 farms of four sub-prefectures of the Abidjan district were analyzed for the presence of *Yersinia*. The two steps enrichment procedure used in this study allowed the isolation of 19 strains (Table 1).

**Table 1:** Samples analyzed for the detection of *Yersinia* strains in animals and humans.

Host		District				*Yersinia*		Total
		Abidjan	Anyama	Bingerville	Songon	Negative	Positive	
Animal	Pig	209	98	306	168	762	19	781
	sheep	69	15	35	59	178	0	178
	Bovine	55	0	22	32	109	0	109
	Rodent	47	0	62	30	139	0	139
	Snail	17	0	23	19	59	0	59
Human	Patients with diarrhea	161	14	93	19	285	2	287
	Patients without diarrhea	87	9	32	11	139	0	139
**Total**		**645**	**136**	**573**	**338**	**1671**	**21**	**1692**

**Fig 1:**
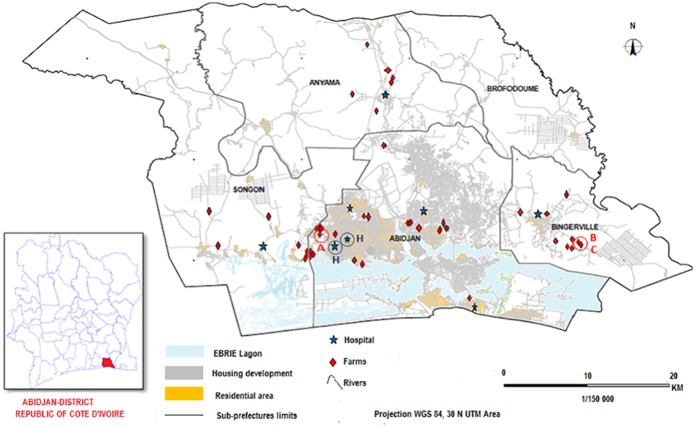
Location of the sampling farms and hospitals in the Abidjan district. Red circles indicate the location of the farms (A, B and C) from which *Yersinia* strains were isolated, and blue circles (H) the location of the hospitals where human cases of yersiniosis were identified.

Seven of them belonged to the non-pathogenic species *Yersinia intermedia*. All 7 *Y*. *intermedia* strains were of biotype 4, but they had different serotypes: 2 were of serotype O:7,8-8-8,19, while the remaining 5 were of serotype O:7,8-8-13-8,19 (S2 Table); indicative of the circulation of different *Y*. *intermedia* strains among pigs in this area. These strains were isolated from 3 farms in 2 sub-prefectures (Bingerville and Abidjan, Fig 1) over a ≈1-year period (S2 Table).

All other 12 strains belonged to the species *Y*. *enterocolitica* and were of bioserotype 4/O:3 (Table 2). These pathogenic *Y*. *enterocolitica* were recovered from pig feces during a period extending from March to August without isolation during the other 6 months of the year, suggesting a periodicity in the carriage of pathogenic *Y*. *enterocolitica* by swine in the Abidjan district. In contrast, some *Y*. *intermedia* strains were isolated in February (S2 Table).

**Table 2:** Characteristics of the *Y*. *enterocolitica* strains isolated from human and animal samples.

Strain #	Host	Bio-type	Serotype	Farm	Sub-prefecture	Phage type	Date of isolation	Virulence genes		
								*ail*	*ystA*	*yadA*
IP35459	Pig	4	O:3	A	Abidjan	XI	2012, August 30	+	+	+
IP35462	Pig	4	O:3	C	Bingerville	VIII	2013, March 21	+	+	+
IP35463	Pig	4	O:3	B	Bingerville	VIII	2013, March 21	+	+	+
IP35464	Pig	4	O:3	C	Bingerville	VIII	2013, April 16	+	+	-
IP35465	Pig	4	O:3	A	Abidjan	XI	2013, April 18	+	+	+
IP35466	Pig	4	O:3	A	Abidjan	XI	2013, May 23	+	+	-
IP35467	Pig	4	O:3	B	Bingerville	XI	2013, May 28	+	+	+
IP35470	Pig	4	O:3	C	Bingerville	VIII	2013, June 25	+	+	-
IP35471	Pig	4	O:3	C	Bingerville	XI	2013, June 25	+	+	+
IP35472	Pig	4	O:3	A	Abidjan	XI	2013, July 9	+	+	+
IP35474	Pig	4	O:3	C	Bingerville	VIII	2013, July 16	+	+	-
IP35475	Pig	4	O:3	C	Bingerville	VIII	2013, August 15	+	+	-
IP35477	Human	4	O:3	NA	Abidjan	XI	2013, April 26	+	+	+
IP35478	Human	4	O:3	NA	Abidjan	XI	2013, September 05	+	+	+

NA: Not applicable

The *Y*. *enterocolitica* strains were isolated from the same 3 farms from which the *Y*. *intermedia* strains were isolated (Table 2). The isolation rate of *Yersinia* strains (pathogenic and non-pathogenic) was similar (8 to 9%) in the 3 farms (S3 Table). Farms B and C were close to each other (1.5 km) in the Bingerville sub-prefecture (Fig 1), arguing for a local circulation of *Y*. *enterocolitica* in this area. Farm A was located in the Abidjan sub-prefecture and was distant by more than 30 km from the other two, indicating the existence of at least two different geographical foci of yersiniosis in the Abidjan district.

### Search for the presence of *Yersinia* in other animal species living in or around the farms

To determine whether pathogenic *Y*. *enterocolitica* strains were circulating among other animal species living in the farms, fecal samples were taken from sheep and bovine and pooled after each visit. None of these samples (178 from cattle and 109 from bovine) were found infected with a *Yersinia* strain (Table 1). Furthermore, 202 rodents (*Rattus*, *Rattus norvegicus* and *Thryonomys swinderianus* (greater cane rat)) and 95 gigantic snails *(Achatina fulica*) living around the farms were captured. Their intestinal content was taken during autopsy and analyzed for the presence of *Yersinia*. No *Yersinia* strains were recovered from these wild animals. These results confirm the preferential association of *Y*. *enterocolitica* 4/O:3 with pigs [44,45]

### Analysis of human stools for the presence of *Yersinia*

Between June 2012 and December 2013, 426 fecal samples were collected from humans in 8 hospitals from the 4 sub-prefectures (Fig 1 and Table 1). They were collected from 287 patients with diarrhea during a childhood diarrhea surveillance program (205 samples), or as part of routine stool cultures in medical microbiology laboratories (82 samples). The 139 other patients presented with digestive disorders (abdominal pain, nausea, vomiting) without diarrhea. Overall the patients aged between 7 months and 55 years with an average of 4 years.

Two *Yersinia* strains were recovered from the 426 human fecal samples analyzed, indicating a prevalence of 0.47% for all patients with intestinal disorders, and of 0.69% for patients with diarrhea. The two patients were infant females aged 1 year 7 months, and 3 years 2 months who presented with diarrhea and fever. They were seen at the university hospital and at the annex of the hospital (2 km away) in the sub-prefecture of Abidjan (Fig 1).

Both clinical strains were pathogenic *Y*. *enterocolitica* 4/O:3 (Table 2).They were isolated in April and September from the 2 patients (Table 2), i.e. at a time of the year where pigs were also found to be carriers of *Y*. *enterocolitica* 4/O:3 strains.

### Phage typing of *Y*. *enterocolitica* 4/O:3 strains

Phage typing is a simple means, routinely used by the French *Yersinia* Reference Laboratory to subgroup 4/O:3 strains. This subtyping method was applied to the Ivorian *Y*. *enterocolitica* strains to determine whether the porcine and human isolates share the same phage type (ΦT). Six strains of pig origin were of ΦT VIII (Table 2), which is by far the most common ΦT among bioserotype 4/O:3 strains worldwide [3,46]. The other 8 strains exhibited ΦT XI (Table 2), which is highly unusual in 4/O:3 strains. These ΦT XI strains were isolated from all 3 farms and from the 2 human patients.

ΦT XI means susceptibility profiles to the 12 lysogenic phages different from the well established ones (VIII, IXa and IXb) for 4/O:3 strains [33]. Actually, a susceptibility or resistance phenotype to each phage was sometimes hard to assign because the growth of ΦT XI strains was heterogeneous within the lysis zone. We often noted a bacterial growth that was less dense in contact to than at distance from the phage drop, or isolated colonies growing within the lysis zone. These suggested mixed bacterial populations composed of colonies susceptible and resistant to each phage.

To test this hypothesis, 3 ΦT XI strains (IP35471, IP35477 and IP35478) were selected and 6 individual colonies from each of them were picked and individually phage typed. Two to 3 susceptibility profiles were observed for each strain (S4 Table), confirming heterogeneity of the bacterial population. Some colonies of strains IP35471 and IP35477 displayed the usual ΦT VIII, arguing for the emergence of variants from this parental phenotype. The 2 other susceptibility profiles were designated ΦT XIa and XIb. ΦT XIa was identified in colonies from the 3 strains, while ΦT XIb colonies were detected in 2 strains (S4 Table). Since a single colony from each biological sample was originally picked and stored, these data show that mutations leading to variable susceptibility or resistance to the set of lysogenic phages occurred in these strains. Altogether these results argue for an unusual propensity of some Ivorian strains to display heterogeneous profiles of resistance to the set of *Y*. *enterocolitica* lysogenic phages.

### Antibiotic susceptibility and virulence markers of the *Y*. *enterocolitica* strains isolated in the Abidjan district

As commonly observed [47,48], the *Y*. *enterocolitica* 4/O:3 strains isolated in our study were resistant to amoxicillin, amoxicillin/clavulanic acid, cefalotin, and ticarcillin. However, they were susceptible to cefoxitin, ceftriaxone, ciprofloxacin, nalidixic acid, trimethoprim, sulphonamide and tetracycline, indicating that they had not acquired any unusual antibiotic resistances.

As expected, all 14 *Y*. *enterocolitica* 4/O:3 strains carried the *ail* and *ystA* chromosomal virulence genes (Table 2), further indicating their pathogenic potential. Only 9 of them (64.3%) harbored the pYV-borne *yadA* gene, in line with the observation that the pYV plasmid is easily lost upon in vitro subculture of pathogenic *Yersinia* [9].

### Genotypic characterization of the *Y*. *enterocolitica* strains isolated

**Fig 2:**
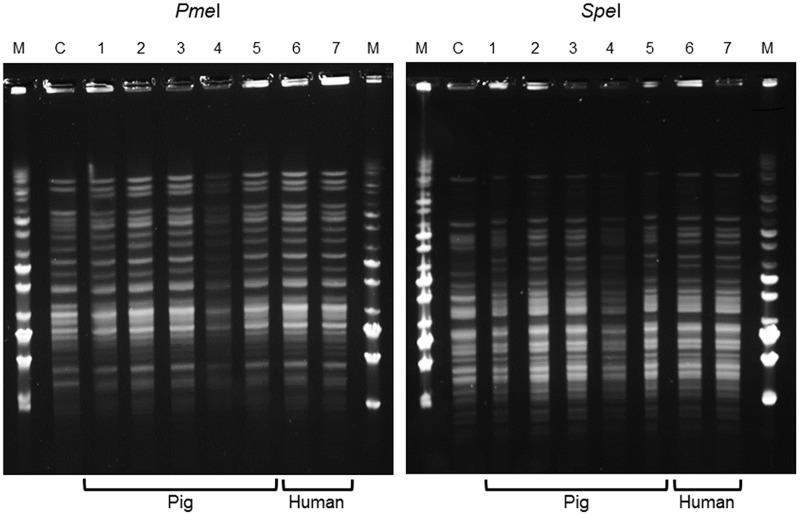
Pulsed Field Gel Electrophoresis patterns of 7 *Y*. *enterocolitica* 4/O:3 strains isolated from animals and humans. C: Control *Y*. *enterocolitica* 4/O:3 strain IP134, M: middle range molecular size marker, 1: IP35470; 2: IP35471; 3: IP35472; 4: IP35474; 5: IP35475; 6: IP35477; 7: IP35478.

PFGE analysis after digestion with *Pme*I and *Spe*I of the 14 *Y*. *enterocolitica* 4/O:3 strains isolated from pigs and humans showed a single profile with each enzyme for all strains analyzed (illustrated in Fig 2). This unique profile differed only slightly from that of an epidemiologically unrelated *Y*. *enterocolitica* 4/O:3 control strain (IP134, isolated in Sweden).

As the discriminatory power of PFGE has recently been shown to be low for pathogenic *Y*. *enterocolitica* [49], the strains were then subjected to MLVA, which has a higher discriminatory power [36,37]. This MLVA analysis based on six loci also gave a unique pattern (7-8-7-8-8-2) for the 14 *Y*. *enterocolitica* strains analyzed. This pattern differed drastically from that of the IP134 control strain (11-2-9-16-6-X), indicating either that the *Y*. *enterocolitica* strains isolated in the Abidjan district are genetically closely related, or that the same strain circulated in the 3 pig farms and in humans.

### Genetic relationships among the Ivorian *Y*. *enterocolitica* strains

To further examine the genetic relatedness of the *Y*. *enterocolitica* 4/O:3 strains isolated from pigs and humans in the Abidjan district over the study period, the genomes of the 14 *Y*. *enterocolitica* strains collected were sequenced. A pair-wise analysis of Single Nucleotide Polymorphism (SNP) showed that the Ivorian strains displayed between 590 and 675 SNPs with the epidemiologically unrelated *Y*. *enterocolitica* 4/O:3 strain IP134 (Table 3). In contrast, the number of SNPs between each pair of Ivorian strains was always <96, indicating that they were genetically much closer to each other than to the epidemiologically unrelated strain. Two isolates (IP35462 and IP35464) had no SNPs between each other. They were both of ΦT VIII and were isolated at one-month interval from the same farm, suggesting that one strain was persisting and isolated twice from farm C. The other strains displayed some degrees of genetic polymorphism (Table 3). These results thus demonstrate that most of the strains that circulate in the pig and human populations in the Abidjan area are distinct from each other.

**Table 3:** Pair-wise analysis of SNPs among Ivorian *Y*. *enterocolitica* strains and an epidemiologically unrelated 4/O:3 isolate.

**Strains**	**IP 134**	**IP 35459**	**IP 35462**	**IP 35463**	**IP 35464**	**IP 35465**	**IP 35466**	**IP 35467**	**IP 35470**	**IP 35471**	**IP 35472**	**IP 35474**	**IP 35475**	**IP 35477**	**IP 35478**
**IP134**	0														
**IP35459**	668	0													
**IP35462**	595	86	0												
**IP35463**	598	86	2	0											
**IP35464**	596	86	0	2	0										
**IP35465**	595	87	5	7	5	0									
**IP35466**	662	42	80	82	80	81	0								
**IP35467**	666	48	86	88	86	85	16	0							
**IP35470**	591	83	11	13	11	10	77	81	0						
**IP35471**	674	70	94	96	94	93	64	68	89	0					
**IP35472**	675	69	93	95	93	94	63	69	90	45	0				
**IP35474**	590	84	12	14	12	13	78	84	3	92	91	0			
**IP35475**	590	83	11	13	11	10	77	81	2	89	90	5	0		
**IP35477**	659	59	81	83	81	80	53	57	76	45	46	79	76	0	
**IP35478**	673	69	93	95	93	92	63	67	88	55	56	91	88	26	0

**Fig 3:**
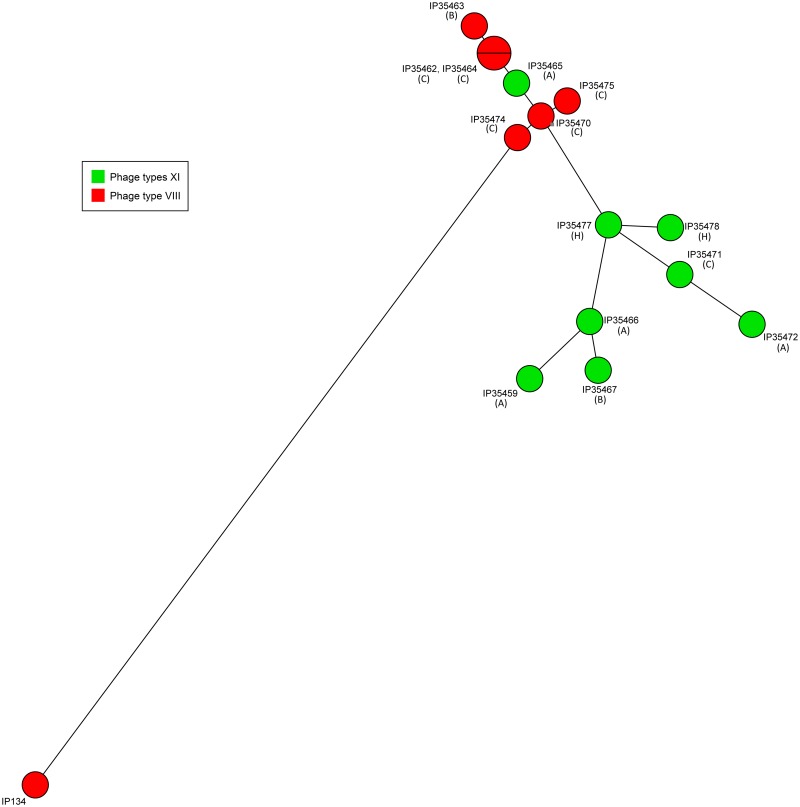
Minimal spanning tree of the *Y*. *enterocolitica* 4/O:3 strains isolated from pigs and humans in the Abidjan district. IP134 is a non-Ivorian *Y*. *enterocolitica* 4/O:3/VIII strain. Letters into brackets indicate the pig farm (A, B, C) or the human (H) origin of the strains.

A minimal spanning tree (MST) based on whole genome SNP analysis was constructed to determine the genetic relatedness between the Ivorian *Y*. *enterocolitica* isolates. This tree further showed that the Ivorian strains were genetically much closer to each other than to the epidemiologically unrelated *Y*. *enterocolitica* 4/O:3/VIII strain IP134 (Fig 3).

The isolates grouped neither according to their geographical location, nor to their date of isolation, arguing for a circulation of strains between the 3 farms, even though farm A was distant from farms B and C. Interestingly, with the exception of strain IP35465, the Ivorian isolates separated into two clusters based on their ΦT (Fig 3). This and the fact that strain IP134 branched with Ivorian strains of ΦT VIII, further strengthen the hypothesis that strains exhibiting the unusual ΦT XI are variants that emerged and gradually diverged from an ancestral ΦT VIII strain.

### Genetic bases for the heterogeneity of the phage susceptibility profiles

The lipopolysaccharide O-antigen has been shown to act as a receptor for various *Y*. *enterocolitica* phages [50]. Although this has not been demonstrated for the set of phages used for *Y*. *enterocolitica* phage typing at the Reference Laboratory, we hypothesized that mutations in the O-antigen gene cluster might have occurred in the ΦT XI branch of the Ivorian isolates and could be responsible for the unusual phage susceptibility patterns observed. However, no polymorphism in the 6.8 kb nucleotide sequence of the O-antigen gene cluster was observed in Ivorian strains, indicating that their unusual susceptibilities to the set of phages are due to other unidentified mutations.

To further understand the genetic bases for the heterogeneity of the phage susceptibility profiles, the genomes of 2 colonies of ΦT XIa (#5 and #6) and of 1 colony of ΦT VIII (#4) from strain IP35471 were sequenced. The aim was to identify genetic modifications that would be common to the 2 ΦT XIa colonies and absent from the ΦT VIII colony, and could thus account for the change in the phage susceptibility profiles. No gene deletions, insertions or mutations (SNPs) having these characteristics were identified among the 3 genomes. However, this analysis revealed an unexpectedly high number of SNPs (9 to 12) between individual colonies of the same strain. To further explore this phenomenon, the number of within-strain SNPs was evaluated on 9 individual colonies from 3 strains of ΦT XI and compared to that of individual colonies from a typical ΦT VIII *Y*. *enterocolitica* 4/O:3 strain (IP33927). While the number of SNPs between colonies of strain IP33927 was always ≤2 (average of 1 SNP), the 3 Ivorian strains of ΦT XI exhibited between 9 and 16 within-colonies SNPs (average of 12 SNPs) (S5 Table), indicating a >10-fold higher mutation rate in these strains.

### Hypermutator phenotype of ΦT XI Ivorian *Y*. *enterocolitica* strains

The higher mutation rate observed in the Ivorian strains of ΦT XI was suggestive of a hypermutator phenotype [51]. To determine whether ΦT XI *Y*. *enterocolitica* Ivorian isolates do have a higher rate of mutations than typical ΦT VIII strains, colonies from 3 ΦT XI strains: IP35471#5 (XIa), IP35477#3 (XIa) and IP35478#4 (XIb) were selected along with strain IP35463 that exhibits the usual ΦT VIII. The capacity to grow on nalidixic and rifampicin agar plates was evaluated for 10 colonies from each of these 4 strains. While very few colonies (≤3) of IP35463 ΦT VIII grew on rifampicin and nalidixic acid agar plates, an average of ≈1000 to 2000 spontaneous Rif^R^ and Nal^R^ mutants were observed in the 3 ΦT XI strains (Table 4). Our results thus demonstrate that some Ivorian *Y*. *enterocolitica* strains have a hypermutator phenotype.

**Table 4:** Average number of spontaneous Rif^R^ and Nal^R^ mutants in colonies with various phage types.

Strain	Phage type	Rif^R^ mutants^a^	Nal^R^ mutants^a^
**IP35463**	VIII	1	3
**IP35471#5**	XIa	1670	2071
**IP35477#3**	XIa	1426	934
**IP35478#4**	XIb	2002	937

^a^ cfu number/10^9^ cfu of the initial inoculum, average of 10 colonies.

A hypermutator phenotype has been linked to mutations in several genes involved in the fidelity of DNA replication, and more particularly in *mutS* [51]. When we looked at this gene in the genome of all 14 Ivorian strains, we observed that the 6 ΦT VIII strains had an intact and identical sequence, while all 8 ΦT XI strains, except IP35465, exhibited a 960 bp deletion at the 3' end of *mutS* (S1A Fig), corresponding to position 935,820 to 936,779 in the reference genome YE1203. This deletion would lead to the synthesis of a protein truncated of more than one third of its normal size (S1B Fig). Therefore the hypermutator phenotype most likely results from a large deletion of the *mutS* gene in some Ivorian isolates.

## Discussion

Diarrheal diseases are a major public health problem in developing countries, with a high infant mortality rate in Africa [52]. Adapted therapeutic measures and control strategies are essential, but cannot be implemented without a proper identification of the etiological agents. Yersiniosis is the third most frequent bacterial disease causing human enteric infections in Europe [10], but reports of this disease are extremely infrequent in developing countries. In West Africa, only few countries reported the isolation of *Yersinia* from clinical cases [11–20], most likely because an active search for these bacteria is not performed. This is supported by the observation that in Nigeria, where studies were carried out to specifically look for this pathogen in human and animal samples, *Yersinia* strains were isolated [15–19]. A major reason for the lack or poor detection of these bacteria is the difficulty to recover them from poly-contaminated samples such as stools, which contain an abundant bacterial flora [53]. Indeed, *Yersinia* strains differ from other enterobacteria by a slower growth rate (48h instead of 24h) and an optimal growth temperature of 28°C instead of 37°C. Therefore, cultures performed under conditions suitable for most enteropathogens are not effective for the recovery of *Yersinia* colonies from polycontaminated biological samples. Specific procedures are required to enhance the isolation rate [53–55], but these procedures are time consuming and costly, and therefore are not performed on a routine basis in most laboratories in West Africa.

There were some indications that pathogenic *Yersinia* are circulating in the swine reservoir in the Abidjan area of Côte d'Ivoire, as a few pathogenic strains of *Y*. *enterocolitica* were isolated from pig carcasses at slaughter houses [30]. In this work we wanted to get an estimate of the number and distribution of *Yersinia*-infected pig farms, and most importantly to determine whether enteric yersiniosis is a cause of human diarrhea in the Abidjan district of Côte d'Ivoire. Using a procedure for the specific isolation of *Yersinia* strains that included several enrichment steps (growth at 25°C, addition of novobiocin, enrichment at 4°C, and growth on CIN agar), we were able to isolate 19 *Yersinia* strains from 781 samples of pig stools collected in 41 farms over 19 months.

Seven of these strains belonged to the non-pathogenic species *Y*. *intermedia*. This species was also previously recovered form pigs at slaughter in the Abidjan region, but the strains had biotypes or serotypes different from those of this study, and they were isolated from other areas [30]. *Y*. *intermedia* strains were also isolated from rectal or tongue swabs of healthy pigs in Nigeria [21,22], suggesting that the environmental conditions in West African countries are favorable for the maintenance of this non-pathogenic species.

The other 12 strains isolated from pig feces were pathogenic *Y*. *enterocolitica*. Pigs are regarded as the major reservoir of enteropathogenic *Y*. *enterocolitica* in most countries worldwide [56]. Although these animals were also found to be carriers of *Yersinia* in the Abidjan district, none of the other cattle sampled within these farms and of the rodents captured in the vicinity of the farms were found infected with *Y*. *enterocolitica*. Snails were also sampled because giant African snails may be abundant around the farms, they are widely consumed as a source of protein, and it was previously shown that they may carry enteropathogenic *Y*. *enterocolitica* for long periods of time [57]. However, none of the 95 snails analyzed were found infected. Therefore our findings support the hypothesis that, as in many other countries, pigs are the main reservoir of enteropathogenic *Y*. *enterocolitica* in the Abidjan district.

According to the 2012 annual report of the Department of Animal Production, over 60% of the national pig production is concentrated in the farms of the Abidjan District. At pig slaughterhouses, meat inspection is limited to a search for macroscopic lesions on the carcasses, without any microbiological investigations. The prevalence of infected pigs at slaughterhouses is usually higher than in farms because samplings are performed on tonsils, which are the most reliable tissue to evaluate the carriage of enteropathogenic *Yersinia* [58], while this cannot be done in live pigs owing to animal welfare. Since excretion of *Y*. *enterocolitica* in the feces is transient, the prevalence of infected pig farms (3/41) in the Abidjan district is thus most likely an underestimation of the risk of human exposure to yersiniosis upon consumption of pork meat.

Although some *Yersinia* strains were previously isolated from human stools in Côte d'Ivoire [29], their species and bioserotype were not determined, so it was not possible to establish whether they were enteropathogenic. Our active search for Y*ersinia* in patients presenting with digestive disorders in the Abidjan district identified two human cases of *Y*. *enterocolitica* infections. This is the first demonstration that yersiniosis is a cause of human diarrhea in this country. These patients were two female infants from the same area (Yopougon). However, they were infected at 4 months interval, indicating that their infection was not caused by the consumption of the same contaminated product.

The 2 human strains had the same bioserotype 4/O:3 as the pig strains. Three strains of this bioserotype were also previously recovered from raw pig samples at slaughterhouse in the Abidjan district [30]. This bioserotype is the most frequently isolated from pigs and human cases in most countries worldwide [8], although 2/O:9 was the predominant bioserotype in *Y*. *enterocolitica* strains isolated from pigs and human cases in the recent years in Nigeria [18,20]. However, since the selective CIN agar we used for the enrichment procedure is inhibitory for some *Y*. *enterocolitica* strains of serotypes O:8 and O:9, and for *Y*. *pseudotuberculosis* [59,60], the possibility that other pathotypes of *Yersinia* circulate in the Abidjan region cannot be excluded.

The frequency of pathogenic *Y*. *enterocolitica* isolated from human stools greatly varies depending on the study, the country, the patients recruited, and the isolation procedures. For instance it was reported to be 0.19% (82/41,848 patients) in Finland [61], 4% (24/600 patients) in Palestine [62], 0.74% (36/4,841 patients) in the USA [63], 0.16% (6/3,784 patients) in UK [64], 0.6% (46/7,090 patients) in Crete [65], 2.46% (267/10,838 patients) in Belgium [66], 0.13% (3/2250 patients) in Canada [67], 0.42% (4/956 patients) in China [68], or 0% in Ireland (0/1,189 patients) [69] and the Netherlands (0/857 patients) [70]. In the Abidjan district, the isolation rate was 0.46% (2/427 patients), and therefore equivalent to or higher than those reported in several European countries (Finland, UK, Ireland and the Netherlands), Canada and China. This demonstrates that, although neglected, *Y*. *enterocolitica* may be a cause of human diarrhea as frequent in Côte d'Ivoire as in European countries.

As usually observed, the *Y*. *enterocolitica* 4/O:3 isolates from the Abidjan district were susceptible to most antibiotics commonly used to treat Gram-negative enteropathogens, and were resistant to penicillin and first and second-generation cephalosporin, due to the presence of the chromosomal *blaA* and *blaB* genes [71,72]. If the diagnosis of yersiniosis is not made, these classes of antibiotics may be used to treat patients, thus leading to treatment failure.

Although pigs and human patients were sampled all year round, pathogenic *Y*. *enterocolitica* strains were only isolated during a period extending from March to September. This period overlaps both the 2 dry seasons (March to May, and August to September) and the rainy season (May to August), with no major temperature variations along the year in the Abidjan district. The periodicity of the *Y*. *enterocolitica* carriage by pigs and consequent human infections may thus have causes independent of the climatic conditions.

The Ivorian *Y*. *enterocolitica* 4/O:3 isolates shared an identical PFGE and MLVA profile, and at the whole genome level they were genetically much closer to each other than to a non-Ivorian isolate. The relative clonality of the strains isolated in the Abidjan district is suggestive of a single import followed by the spread and diversification of the introduced strain. However, we noted that the *Y*. *enterocolitica* isolates could be phenotypically subdivided into ΦT VIII and ΦT XI, the latter being highly unusual. Indeed, in the collection of the French *Yersinia* Reference Laboratory, which includes numerous strains of worldwide origins, 7,853 out of the 8,295 *Y*. *enterocolitica* 4/O:3 that were phage typed were of ΦT VIII (94.7%), while only 25 of them (0.3%) were of ΦT XI. This unusual ΦT and the existence of ΦT subgroups within colonies of the same strain prompted us to perform a whole genome analysis. This analysis revealed that ΦT XI isolates had actually a hypermutator phenotype most likely caused by a large deletion at the 3' end of the *mutS* gene. In *Escherichia coli*, large deletions removing the 3' end of *mutS* have also been shown to cause an excessive rate of point mutations [73]. *mutS* is involved in DNA mismatch repair that ensures the fidelity of replication of the bacterial chromosome [74]. The variety of ΦT observed in colonies harboring the *mutS* deletion thus probably results from random mutations occurring at higher frequencies in various genes, some of them used by phages to enter or kill their bacterial hosts. Of note, only one ΦT XI strain (IP35465) carried an intact *mutS* gene, and this is the only ΦT XI isolate that grouped with ΦT VIII strains in the minimal spanning tree. It is therefore likely that this strain harbored a mutation in a gene conferring resistance to some phages, but because its genome was not prone to multiple mutations, it was genetically closer to the non-mutator strains of ΦT VIII.

Since the deletion of *mutS* was identical in all Ivorian isolates, this event probably occurred once in an ancestral strain from which the other *mutS* strains derived. The ability to rapidly expand mutant cell types is a clear advantage for pathogenic organisms to evade host defenses and drugs, and to adapt to stresses and changing environments [73]. The longer branches between Ivorian strains in the hypermutator cluster, as compared to the non-hypermutator cluster in the minimal spanning tree, are indicative of a faster genetic diversification of the mutator strains. This may thus lead to the expansion of pathogenic *Y*. *enterocolitica* strains with new phenotypes that may increase their capacity to multiply in their animal host or to cause severe infections in humans. Mutations leading to resistances to several classes of antibiotics may also arise at higher frequencies. Since the hypermutator phenotype is most likely caused by a large deletion of *mutS*, the reversion to a non-mutator phenotype is now hardly possible in these strains.

In conclusion, this study demonstrated that pathogenic *Y*. *enterocolitica* are circulating in the pig reservoir in Côte d'Ivoire and are causing human infections with a prevalence comparable to that of some European countries. The paucity of reports of this infection in African countries is most likely attributable to a lack of active detection rather than to an absence of the microorganism. The identification of hypermutator strains circulating in the pig reservoir and in humans may be of concern as these strains may acquire at a faster rate selective advantages that may increase their fitness, pathogenesis or resistance to commonly used treatments.

## Supporting Information

S1 Fig (PDF)

S1 Table Primers used for PCR amplification of virulence genes. (DOC)

S2 Table Characteristics of the *Y*. *intermedia* strains isolated from pigs. (DOC)

S3 Table Frequency of isolation of *Yersinia* strains in different farms. (DOC)

S4 Table Susceptibility profile of individual colonies from 3 *Y*. *enterocolitica* 4/O:3 strains to the set of lysogenic phages. (DOC)

S5 Table Pair-wise analysis of SNPs among individual colonies of phage type VIII and XI strains. (DOC)
