# Supplementary material for: Yersinia enterocolitica, a Neglected Cause of Human Enteric Infections in Côte d’Ivoire
Source: PLoS Negl Trop Dis. 2017 Jan 12;11(1):e0005216. doi: 10.1371/journal.pntd.0005216 (PMC5230755; doi:10.1371/journal.pntd.0005216)
Supplement: S1 Fig — (PDF) [file pntd.0005216.s001.pdf]

Figure S1.

A. Nucleotide alignment of the *mutS* sequence from non-hypermutator (wild type) and hypermutator (truncated) *Y. enterocolitica* strains from Cote d'Ivoire

```
wild-type_MutS      ATGAATAATACTGATAAGCTAGATTCACACACCCCAATGATGCAGCAGTATCTTCGGCTC      60
truncated_MutS      ATGAATAATACTGATAAGCTAGATTCACACACCCCAATGATGCAGCAGTATCTTCGGCTC      60
                    ************************************************************

wild-type_MutS      AAAGCCCAACATCCTGAAATACTCCTGTTTTATCGGATGGGAGATTTTTATGAGCTGTTC      120
truncated_MutS      AAAGCCCAACATCCTGAAATACTCCTGTTTTATCGGATGGGAGATTTTTATGAGCTGTTC      120
                    ************************************************************

wild-type_MutS      TACAGTGATGCCAAACGCGCGTCCCAGTTATTGGATATCTCACTGACAAAACGGGGGGCT      180
truncated_MutS      TACAGTGATGCCAAACGCGCGTCCCAGTTATTGGATATCTCACTGACAAAACGGGGGGCT      180
                    ************************************************************

wild-type_MutS      TCCGCCGGTGAACCTATTCCTATGGCGGGTGTTCCCTATCATTCGATAGAAAACTATCTG      240
truncated_MutS      TCCGCCGGTGAACCTATTCCTATGGCGGGTGTTCCCTATCATTCGATAGAAAACTATCTG      240
                    ************************************************************

wild-type_MutS      GCAAAGCTGGTTCAATTGGGTGAGTCAGCGGCTATCTGTGAGCAAATCGGTGATCCGGCT      300
truncated_MutS      GCAAAGCTGGTTCAATTGGGTGAGTCAGCGGCTATCTGTGAGCAAATCGGTGATCCGGCT      300
                    ************************************************************

wild-type_MutS      ACCAGCAAAGGGCCGGTTGAACGTAAAGTCGTGCGAATAGTCACCCCCGGTACCGTGAGT      360
truncated_MutS      ACCAGCAAAGGGCCGGTTGAACGTAAAGTCGTGCGAATAGTCACCCCCGGTACCGTGAGT      360
                    ************************************************************

wild-type_MutS      GACGAGGCCTTACTACAAGAGCGCCAGGATAATCTTCTGGCCGCCATCTGGCAGGATACG      420
truncated_MutS      GACGAGGCCTTACTACAAGAGCGCCAGGATAATCTTCTGGCCGCCATCTGGCAGGATACG      420
                    ************************************************************

wild-type_MutS      CGCGGATTTGGTTATGCAACGCTGGATATCAGTTCAGGCCGCTTTAGAGTTGCAGAACCC      480
truncated_MutS      CGCGGATTTGGTTATGCAACGCTGGATATCAGTTCAGGCCGCTTTAGAGTTGCAGAACCC      480
                    ************************************************************

wild-type_MutS      GCAGACCTTGAAACTATGGCTGCCGAGTTACAACGTACCAATCCCGCAGAGTTACTTTAT      540
truncated_MutS      GCAGACCTTGAAACTATGGCTGCCGAGTTACAACGTACCAATCCCGCAGAGTTACTTTAT      540
                    ************************************************************

wild-type_MutS      CCAGAAAACTTCGAGCAAATGTCGTTGATCGAACATCGACATGGCTTACGCCGCCGCCCA      600
truncated_MutS      CCAGAAAACTTCGAGCAAATGTCGTTGATCGAACATCGACATGGCTTACGCCGCCGCCCA      600
                    ************************************************************

wild-type_MutS      TTATGGGAGTTTGAGCTGGAAACAGCCAAACAGCAGTTAAACCTGCAATTCGGCACCCGC      660
truncated_MutS      TTATGGGAGTTTGAGCTGGAAACAGCCAAACAGCAGTTAAACCTGCAATTCGGCACCCGC      660
                    ************************************************************

wild-type_MutS      GATTTAATCGGTTTTGGTGTCGAACAAGCCCATCAGGCACTGCGGGCTGCGGGCTGCCTG      720
truncated_MutS      GATTTAATCGGTTTTGGTGTCGAACAAGCCCATCAGGCACTGCGGGCTGCGGGCTGCCTG      720
                    ************************************************************

wild-type_MutS      CTGCAATATGTCAAAGATACCCAGCGCACCTCCCTACCCCATATCCGTGGCTTGACCATG      780
truncated_MutS      CTGCAATATGTCAAAGATACCCAGCGCACCTCCCTACCCCATATCCGTGGCTTGACCATG      780
                    ************************************************************

wild-type_MutS      GAGCGTCAGCAAGATGGCATTGTTATGGATGCTGCGACCCGTCGTAATCTTGAACTGACG      840
truncated_MutS      GAGCGTCAGCAAGATGGCATTGTTATGGATGCTGCGACCCGTCGTAATCTTGAACTGACG      840
                    ************************************************************

wild-type_MutS      CAGAATTTATCGGGTGGAACGGAAAACACGCTGGCTGCGATCCTCGATTGCACGGTAACC      900
truncated_MutS      CAGAATTTATCGGGTGGAACGGAAAACACGCTGGCTGCGATCCTCGATTGCACGGTAACC      900
                    ************************************************************

wild-type_MutS      GCTATGGGCAGCCGCATGTTGAAACGCTGGCTACATATGCCAATCCGTGACACCAAAGTG      960
truncated_MutS      GCTATGGGCAGCCGCATGTTGAAACGCTGGCTACATATGCCAATCCGTGACACCAAAGTG      960
                    ************************************************************
```

```
wild-type_MutS      TTGACTGATCGCCAGCAAGCCATTGGTGGTCTGCAAGAGATTACCGCTGAACTGCAAACT      1020
truncated_MutS      TTGACTGATCGCCAGCAAGCCATTGGTGGTCTGCAAGAGATTACCGCTGAACTGCAAACT      1020
                    ************************************************************

wild-type_MutS      CCACTGCGTCAGGTCGGGGATTTAGAACGTATTTTGGCACGACTGGCACTGCGAACCGCT      1080
truncated_MutS      CCACTGCGTCAGGTCGGGGATTTAGAACGTATTTTGGCACGACTGGCACTGCGAACCGCT      1080
                    ************************************************************

wild-type_MutS      CGCCCGAGAGATTTGGCAAGAATGCGGCATGCATTTCAGCAACTACCTGAGATTCATCGT      1140
truncated_MutS      CGCCCGAGAGATTTGGCAAGAATGCGGCATGCATTTCAGCAACTACCTGAGATTCATCGT      1140
                    ************************************************************

wild-type_MutS      TTATTGCAGCCAGTGAATGTTCCCCATATCCAAAATTTACTGTCACAAGTCGGCCAGTTT      1200
truncated_MutS      TTATTGCAGCCAGTGAATGTTCCCCATATCCAAAATTTACTGTCACAAGTCGGCCAGTTT      1200
                    ************************************************************

wild-type_MutS      GATGAATTACAAGATTTACTGGAACGCGCAATTGTTGAAACGCCGCCAGTATTGGTACGA      1260
truncated_MutS      GATGAATTACAAGATTTACTGGAACGCGCAATTGTTGAAACGCCGCCAGTATTGGTACGA      1260
                    ************************************************************

wild-type_MutS      GATGGTGGCGTCATCGCCCCTGGCTACAATGCAGAATTAGACGAATGGCGGGCGCTGGCT      1320
truncated_MutS      GATGGTGGCGTCATCGCCCCTGGCTACAATGCAGAATTAGACGAATGGCGGGCGCTGGCT      1320
                    ************************************************************

wild-type_MutS      GATGGTGCAACCGATTATCTCGACCGGTTGGAAATCCGTGAACGCGAAAAACTGGGTCTG      1380
truncated_MutS      GATGGTGCAACCGATTATCTCGACCGGTTGGAAATCCGTGAACGCGAAAAACTGGGTCTG      1380
                    ************************************************************

wild-type_MutS      GATACCCTAAAAGTCGGTTTTAATGGTGTTCATGGCTATTACATTCAGGTTAGCCGTGGT      1440
truncated_MutS      GATACCCTAAAAGTCGGTTTTAATGGTGTTCATGGCTATTACATTCAGGTTAGCCGTGGT      1440
                    ************************************************************

wild-type_MutS      CAGAGCCATCTGGTACCGATTCATTATGTTCGCAGGCAAACGTTGAAGAATGCCGAGCGC      1500
truncated_MutS      CAGAGCCATCTGGTACCGATTCATTATGTTCGCAGGCAAACGTTGAAGAATGCCGAGCGC      1500
                    ************************************************************

wild-type_MutS      TACATCATTCCAGAGCTGAAAGAGTATGAAGACAAAGTTCTGACCTCGAAAGGCAAGGCT      1560
truncated_MutS      TACATCATTCCAGAGCTGAAAGAGTATGAAGACAAAGTTCTGACCTCGAAAGGCAAGGCT      1560
                    ************************************************************

wild-type_MutS      TTGGCGATTGAAAAAGGTTTGTACGAAGAAATTTTCGATCTGCTGTTGCCGCATCTACCT      1620
truncated_MutS      TTGGCGATTGAAAAAGGTTTGTACGAAGAAATTTTC------------------------      1596
                    ************************************

wild-type_MutS      GAGTTACAAACCAGCGCCAATGCACTAGCCGAACTTGATGTTTTGGCAAATCTGGCCGAA      1680
truncated_MutS      ------------------------------------------------------------      1596

wild-type_MutS      AGAGCCGAAACACTCAGCTACAACTGCCCTGTCCTGAGTGATAAACCGGGGATCAAAATT      1740
truncated_MutS      ------------------------------------------------------------      1596

wild-type_MutS      ACTGGCGGCCGTCATCCGGTAGTGGAGCAGGTGCTCAGTGAGCCTTTTATTTCTAACCCG      1800
truncated_MutS      ------------------------------------------------------------      1596

wild-type_MutS      CTGACACTCTCACCTCAACGGCGAATGCTGATCATTACTGGCCCGAATATGGGCGGTAAA      1860
truncated_MutS      ------------------------------------------------------------      1596

wild-type_MutS      AGTACCTATATGCGCCAAACCGCATTGATAGTCTTACTGGCACATATGGGCAGCTACGTT      1920
truncated_MutS      ------------------------------------------------------------      1596

wild-type_MutS      CCTGCAGACCAAGCCACTATTGGGCCAGTTGACCGTATCTTTACTCGAGTCGGTGCTGCT      1980
truncated_MutS      ------------------------------------------------------------      1596

wild-type_MutS      GACGACCTGGCATCCGGCCGCTCAACCTTTATGGTCGAAATGACAGAAACGGCCAATATT      2040
truncated_MutS      ------------------------------------------------------------      1596
```

```
wild-type_MutS     CTGCATAATGCCACCGAACAAAGTTTGGTATTAATGGATGAAATTGGTCGCGGCACATCA     2100
truncated_MutS     ------------------------------------------------------------     1596

wild-type_MutS     ACCTATGATGGCTTGTCATTAGCTTGGGCTTGCGCTGAAAATCTGGCTAGCCGTATCAAA     2160
truncated_MutS     ------------------------------------------------------------     1596

wild-type_MutS     GCCATGACGCTATTTGCTACTCATTACTTTGAGCTAACGACCCTGCCAGAAAAAATGGAG     2220
truncated_MutS     ------------------------------------------------------------     1596

wild-type_MutS     GGGGTGGTCAATGTTCATCTTGATGCATTGGAACATGGCGAAACCATCGCCTTTATGCAC     2280
truncated_MutS     ------------------------------------------------------------     1596

wild-type_MutS     AGTGTGCAAGATGGTGCGGCGAGTAAAAGTTATGGTTTAGCTGTTGCAGCCTTGGCCGGT     2340
truncated_MutS     ------------------------------------------------------------     1596

wild-type_MutS     GTGCCACGGGATGTGATTAAGCGCGCACGGCAAAAACTGAAAGAGCTGGAATCGTTGTCG     2400
truncated_MutS     ------------------------------------------------------------     1596

wild-type_MutS     AACAATGCAGCGGCAAGCAAAATTGATGGCTCGCAACTCACGTTGCTAAATGAAGAGGTA     2460
truncated_MutS     ------------------------------------------------------------     1596

wild-type_MutS     TCGCCAGCAGTGGAAGCCTTAGAGTCATTGGATCCAGACTCCCTATCACCACGTCAGGCG     2520
truncated_MutS     ------------------------------------------------------------     1596

wild-type_MutS     CTTGAGTGGATTTATCGCCTGAAAAACATGGTGTAA 2556
truncated_MutS     ------------------------------------ 1596
```

B. Amino acid alignment of the predicted MutS product from non-hypermutator (wild type) and hypermutator (truncated) *Y. enterocolitica* strains from Cote d'Ivoire

```
wild-type_MutS      MNNTDKLDSHTPMMQQYLRLKAQHPEILLFYRMGDFYELFYSDAKRASQLLDISLTKRGA      60
truncated_MutS      MNNTDKLDSHTPMMQQYLRLKAQHPEILLFYRMGDFYELFYSDAKRASQLLDISLTKRGA      60
                    ************************************************************

wild-type_MutS      SAGEPIPMAGVPYHSIENYLAKLVQLGESAAICEQIGDPATSKGPVERKVVRIVTPGTVS      120
truncated_MutS      SAGEPIPMAGVPYHSIENYLAKLVQLGESAAICEQIGDPATSKGPVERKVVRIVTPGTVS      120
                    ************************************************************

wild-type_MutS      DEALLQERQDNLLAAIWQDTRGFGYATLDISSGRFRVAEPADLETMAAELQRTNPAELLY      180
truncated_MutS      DEALLQERQDNLLAAIWQDTRGFGYATLDISSGRFRVAEPADLETMAAELQRTNPAELLY      180
                    ************************************************************

wild-type_MutS      PENFEQMSLIEHRHGLRRRPLWEFELETAKQQLNLQFGTRDLIGFGVEQAHQALRAAGCL      240
truncated_MutS      PENFEQMSLIEHRHGLRRRPLWEFELETAKQQLNLQFGTRDLIGFGVEQAHQALRAAGCL      240
                    ************************************************************

wild-type_MutS      LQYVKDTQRTSLPHIRGLTMERQQDGIVMDAATRRNLELTQNLSGGTENTLAAILDCTVT      300
truncated_MutS      LQYVKDTQRTSLPHIRGLTMERQQDGIVMDAATRRNLELTQNLSGGTENTLAAILDCTVT      300
                    ************************************************************

wild-type_MutS      AMGSRMLKRWLHMPIRDTKVLTDRQQAIGGLQEITAELQTPLRQVGDLERILARLALRTA      360
truncated_MutS      AMGSRMLKRWLHMPIRDTKVLTDRQQAIGGLQEITAELQTPLRQVGDLERILARLALRTA      360
                    ************************************************************

wild-type_MutS      RPRDLARMRHAFQQLPEIHRLLQPVNVPHIQNLLSQVGQFDELQDLLERAIVETPPVLVR      420
truncated_MutS      RPRDLARMRHAFQQLPEIHRLLQPVNVPHIQNLLSQVGQFDELQDLLERAIVETPPVLVR      420
                    ************************************************************

wild-type_MutS      DGGVIAPGYNAELDEWRALADGATDYLDRLEIREREKLGLDTLKVGFNGVHGYYIQVSRG      480
truncated_MutS      DGGVIAPGYNAELDEWRALADGATDYLDRLEIREREKLGLDTLKVGFNGVHGYYIQVSRG      480
                    ************************************************************

wild-type_MutS      QSHLVPIHYVRRQTLKNAERYIIPELKEYEDKVLTSKGKALAIEKGLYEEIFDLLLPHLP      540
truncated_MutS      QSHLVPIHYVRRQTLKNAERYIIPELKEYEDKVLTSKGKALAIEKGLYEEIF--------      532
                    ****************************************************

wild-type_MutS      ELQTSANALAELDVLANLAERAETLSYNCPVLSDKPGIKITGGRHPVVEQVLSEPFISNP      600
truncated_MutS      ------------------------------------------------------------      532


wild-type_MutS      LTLSPQRRMLIITGPNMGGKSTYMRQTALIVLLAHMGSYVPADQATIGPVDRIFTRVGAA      660
truncated_MutS      ------------------------------------------------------------      532


wild-type_MutS      DDLASGRSTFMVEMTETANILHNATEQSLVLMDEIGRGTSTYDGLSLAWACAENLASRIK      720
truncated_MutS      ------------------------------------------------------------      532


wild-type_MutS      AMTLFATHYFELTTLPEKMEGVVNVHLDALEHGETIAFMHSVQDGAASKSYGLAVAALAG      780
truncated_MutS      ------------------------------------------------------------      532


wild-type_MutS      VPRDVIKRARQKLKELESLSNNAAASKIDGSQLTLLNEEVSPAVEALESLDPDSLSPRQA      840
truncated_MutS      ------------------------------------------------------------      532


wild-type_MutS      LEWIYRLKNMV*      851
truncated_MutS      ------------      532
```
