# Supplementary material for: Yersinia enterocolitica, a Neglected Cause of Human Enteric Infections in Côte d’Ivoire
Source: PLoS Negl Trop Dis. 2017 Jan 12;11(1):e0005216. doi: 10.1371/journal.pntd.0005216 (PMC5230755; doi:10.1371/journal.pntd.0005216)
Supplement: S1 Table — (DOC) [file pntd.0005216.s002.doc]

**Table S1. Primers used for PCR amplification of virulence genes.**

| **Genes** | **Primers** | **Sequence (5’ to 3’)** | **Size**  **(bp)** | **Annealing**  **T (°C)** | **References** |
| --- | --- | --- | --- | --- | --- |
| *ail* | *ail*-F  *ail*-R | TAATGTGTACGCTGCGAG  GACGTCTTACTTGCACTG | 351 | 57 | [1] |
| *ystA* | ystA-F  ystA-R | AATGCTGTCTTCATTTGGAGC  ATCCCAATCACTACTGACTTC | 145 | 60 | [2] |
| *yadA* | *yadA*-F  *yadA*-R | CTTCAGATACTGGTGTCGCTGT  ATGCCTGACTAGAGCGATATCC | 849 | 60 | [1] |

1. Thoerner P, Bin Kingombe CI, Bogli-Stuber K, Bissig-Choisat B, Wassenaar TM, et al. (2003) PCR detection of virulence genes in *Yersinia enterocolitica* and *Yersinia pseudotuberculosis* and investigation of virulence gene distribution. Appl Environ Microbiol 69: 1810-1816.

2. Ibrahim A, Liesack W, Griffiths MW, Robins-Browne RM (1997) Development of a highly specific assay for rapid identification of pathogenic strains of *Yersinia enterocolitica* based on PCR amplification of the *Yersinia* heat-stable enterotoxin gene (*yst*). J Clin Microbiol 35: 1636-1638.
