# Supplementary material for: Yersinia enterocolitica, a Neglected Cause of Human Enteric Infections in Côte d’Ivoire
Source: PLoS Negl Trop Dis. 2017 Jan 12;11(1):e0005216. doi: 10.1371/journal.pntd.0005216 (PMC5230755; doi:10.1371/journal.pntd.0005216)
Supplement: S2 Table — (DOC) [file pntd.0005216.s003.doc]

**Table S2. Characteristics of the *Y. intermedia* strains isolated from pigs**

| **Strain #** | **Biotype** | **Sérotype** | **Date of isolation** | **Farm** | **Subprefecture** |
| --- | --- | --- | --- | --- | --- |
| IP35458 | 4 | 7,8-8-13-8,19 | 2012, July 17 | A | Abidjan |
| IP35460 | 4 | 7,8-8-13-8,19 | 2013, February 14 | B | Bingerville |
| IP35461 | 4 | 7,8-8-13-8,19 | 2013, February 14 | B | Bingerville |
| IP35468 | 4 | 7,8-8-13-8,19 | 2013, May 28 | C | Bingerville |
| IP35469 | 4 | 7,8-8-13-8,19 | 2013, June 18 | A | Abidjan |
| IP35473 | 4 | 7,8-8-8,19 | 2013, July 16 | C | Bingerville |
| IP35476 | 4 | 7,8-8-8,19 | 2013, August 15 | B | Bingerville |
