# Supplementary material for: Yersinia enterocolitica, a Neglected Cause of Human Enteric Infections in Côte d’Ivoire
Source: PLoS Negl Trop Dis. 2017 Jan 12;11(1):e0005216. doi: 10.1371/journal.pntd.0005216 (PMC5230755; doi:10.1371/journal.pntd.0005216)
Supplement: S3 Table — (DOC) [file pntd.0005216.s004.doc]

**Table S3: Frequency of isolation of *Yersinia*strains in different farms**

| **Farm** | **Total samples analyzed**  **(% of total)** | **Number of *Y. enterocolitica*** | **Number of *Y. intermedia*** | **Total number of *Yersinia*strains**  **(% in each farm)** |
| --- | --- | --- | --- | --- |
| A | 64 (8.2) | 4 | 2 | 6 (9.4) |
| B | 64 (8.2) | 2 | 3 | 5 (7.8) |
| C | 85 (10.7) | 6 | 2 | 8 (9.4) |
| 38 other farms | 568 (72.7) | 0 | 0 | 0 (0.0) |
| **Total** | **781 (100)** | **12** | **7** | **19 (2.4)** |
