# Supplementary material for: Yersinia enterocolitica, a Neglected Cause of Human Enteric Infections in Côte d’Ivoire
Source: PLoS Negl Trop Dis. 2017 Jan 12;11(1):e0005216. doi: 10.1371/journal.pntd.0005216 (PMC5230755; doi:10.1371/journal.pntd.0005216)
Supplement: S4 Table — (DOC) [file pntd.0005216.s005.doc]

**Table S4. Susceptibility profile of individual colonies from 3 *Y. enterocolitica* 4/O:3 strains to the set of lysogenic phages**

| **Strain** | **Colony #** | **Phage** | | | | | | | | | | | | **Phage type** |
| --- | --- | --- | --- | --- | --- | --- | --- | --- | --- | --- | --- | --- | --- | --- |
|  |  | **a** | **b** | **c** | **d** | **e** | **f** | **g** | **h** | **i** | **j** | **k** | **l** |  |
| **IP35471** | **1** | S | S | S | S | S | S | S | S | S | S | S | R | VIII |
|  | **2** | S | S | S | S | S | S | S | S | S | R | S | R | XIa |
|  | **3** | S | S | S | S | S | S | S | S | S | S | S | R | VIII |
|  | **4** | S | S | S | S | S | S | S | S | S | S | S | R | VIII |
|  | **5** | S | S | S | S | S | S | S | S | S | R | S | R | XIa |
|  | **6** | S | S | S | S | S | S | S | S | S | R | S | R | XIa |
| **IP35477** | **1** | R | R | R | R | R | R | R | R | S | R | R | R | XIb |
|  | **2** | R | R | R | R | R | R | R | R | S | R | R | R | XIb |
|  | **3** | S | S | S | S | S | S | S | S | S | R | S | R | XIa |
|  | **4** | S | S | S | S | S | S | S | S | S | S | S | R | VIII |
|  | **5** | S | S | S | S | S | S | S | S | S | S | S | R | VIII |
|  | **6** | R | R | R | R | R | R | R | R | S | R | R | R | XIb |
| **IP35478** | **1** | S | S | S | S | S | S | S | S | S | R | S | R | XIa |
|  | **2** | R | R | R | R | R | R | R | R | S | R | R | R | XIb |
|  | **3** | R | R | R | R | R | R | R | R | S | R | R | R | XIb |
|  | **4** | R | R | R | R | R | R | R | R | S | R | R | R | XIb |
|  | **5** | S | S | S | S | S | S | S | S | S | R | S | R | XIa |
|  | **6** | S | S | S | S | S | S | S | S | S | R | S | R | XIa |

S: susceptibility to the corresponding phage; R: resistance to the corresponding phage
