# Supplementary material for: Yersinia enterocolitica, a Neglected Cause of Human Enteric Infections in Côte d’Ivoire
Source: PLoS Negl Trop Dis. 2017 Jan 12;11(1):e0005216. doi: 10.1371/journal.pntd.0005216 (PMC5230755; doi:10.1371/journal.pntd.0005216)
Supplement: S5 Table — (DOC) [file pntd.0005216.s006.doc]

**Table S5. Pair-wise analysis of SNPs among individual colonies of phage type VIII and XI strains**

**IP33927 (4/O:3/VIII)**

| **Colony** | **#1** | **#2** | **#3** | **#4** |
| --- | --- | --- | --- | --- |
| **#1** | 0 |  |  |  |
| **#2** | 2 | 0 |  |  |
| **#3** | 2 | 0 | 0 |  |
| **#4** | 0 | 2 | 2 | 0 |

**IP35471 (4/O:3/XI)**

| **Colony** | **#3** | **#4** | **#5** | **#6** |
| --- | --- | --- | --- | --- |
| **#3** | 0 |  |  |  |
| **#4** | 16 | 0 |  |  |
| **#5** | 13 | 11 | 0 |  |
| **#6** | 14 | 12 | 9 | 0 |

**IP35477 (4/O:3/XI)**

| **Colony** | **#2** | **# 3** | **#4** |
| --- | --- | --- | --- |
| **#2** | 0 |  |  |
| **#3** | 13 | 0 |  |
| **#4** | 11 | 12 | 0 |

**IP35478 (4/O:3/XI)**

| **Colony** | **#4** | **#5** |
| --- | --- | --- |
| **#4** | 0 |  |
| **#5** | 12 | 0 |
